# Controlling the Optoelectronic Properties of the Non‐Fullerene Acceptor Y6 via p‐Doping

**DOI:** 10.1002/smll.74397

**Published:** 2026-07-08

**Authors:** Nikita A. Shumilov, Aditi Kumar, Paul A. Hume, Michael B. Price, Nathaniel J. L. K. Davis, Justin M. Hodgkiss

**Affiliations:** ^1^ School of Chemical and Physical Sciences Victoria University of Wellington Wellington New Zealand; ^2^ The Dodd‐Walls Centre for Photonic and Quantum Technologies University of Otago Dunedin New Zealand; ^3^ The MacDiarmid Institute for Advanced Materials and Nanotechnology Wellington New Zealand; ^4^ School of Chemistry University of Bristol Bristol UK

**Keywords:** acceptor, bilayer, dielectricdoping, exciton, heterojunction, materials science, optoelectronics, organic semiconductor, organic solar cell

## Abstract

Historically, the low dielectric constants in organic semiconductors necessitated the need for a molecular heterojunction to split excitons into charges, limiting the performance of organic photovoltaic devices. Recently, non‐fullerene acceptors (NFAs) with relatively high dielectric constants have shown the ability to create charge transfer states, and even the potential for charges, without the need for a heterojunction. Despite this advancement, problems such as charge trapping and recombination still hinder the efficiencies of organic photovoltaic devices. One of the potential strategies to reduce these losses is via p‐doping control, which may help to suppress trap‐assisted recombination. Understanding how doping of an NFA impacts its optoelectronic properties is key to developing highly efficient bulk‐heterojunction and bilayer devices. In this work, we focused on the influence of doping on device operational properties in Y6‐based single‐component solar cells with different concentrations of p‐dopant trityl tetrakis(pentafluorophenyl) borate (TrTPFB). We demonstrate how the addition of TrTPFB in Y6 shows the signs of trap filling, reduces the influence of complex recombination mechanisms, increases the short‐circuit current, and improves the overall efficiency of single‐component solar cells. The results reveal the potential of using p‐doping control of NFAs in heterojunction and bilayer solar cells.

## Graphical abstract

The addition of TrTPFB to non‐fullerene acceptor Y6 is shown to significantly increase photocurrent generation and performance in single‐component solar cells. The findings reveal that doping with TrTPFB can potentially help to fill trap states and reduce recombination, providing new insight into how doping control can improve the performance of organic solar cells.

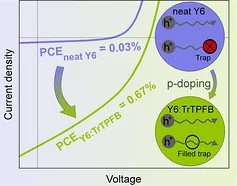

## 1 Introduction

Organic solar cells (OSCs) based on non‐fullerene acceptors (NFAs) have become a promising path to efficient and sustainable solar energy [1]. The features of solution‐processed organic electronics enable the development of semi‐transparent and flexible solar cells, showing potential for applications such as solar windows, wearable technologies, and more [2, 3, 4, 5, 6, 7, 8, 9, 10]. Fused ring electron acceptors are a class of NFAs with high exciton diffusion lengths (20‐47 nm), improved light absorption, and solubility, which have ultimately led to an increase in organic photovoltaic (OPV) performance [11]. These recent advancements in OPVs have resulted in power conversion efficiencies surpassing 20%, driving progress toward more efficient and sustainable renewable energy solutions [12].

Previously, OPV efficiencies have been limited by the need for a molecular heterojunction to split excitons into charges. However, NFAs with relatively high dielectric constants, such as Y6, have recently emerged, where light can potentially generate charges without a heterojunction [13, 14, 15, 16]. NFAs like Y6 have a modest exciton binding energy of around 200 meV to 350 meV and can directly transform excitons into charge transfer states, which split into free charges, improving the yield of intrinsic photocharges in neat films [13, 14, 15, 16]. This discovery opens up the possibility of using simplified single‐component architectures for OPVs. For example, a Y6 homojunction OPV introducing a secondary hole transport layer recently achieved an efficiency of 2.57% [17].

However, along with the difficulty in splitting apart the charges, the short‐lived nature of charges in NFAs is a significant problem and remains one of the key challenges in improving the performance of these new types of OPVs [13]. The doping of organic semiconductors offers a strategy to potentially diminish trap‐assisted monomolecular, interfacial recombination, fast bimolecular recombination, and to improve charge transport [18, 19]. Doping of NFAs can enhance the optoelectronic properties of OPVs and reduce energy losses in a way similar to inorganic doping, but the absence of a covalently bonded host‐dopant system in organic semiconductors complicates the doping process [20]. Unlike inorganic doping processes, the doping of organic molecules requires high concentrations of dopant on the order of 1 per cent to achieve OPV performance improvements [20, 21]. However, these excessively high dopant concentrations may affect the structure of an active material, causing disorder and excess trap state formation in the crystalline films [20, 21, 22]. On the other hand, utilising low concentrations of a dopant in organic semiconductors has been shown to fill trap states and increase device efficiency, but this process requires highly efficient dopants [18, 23, 24]. The study of doping control in organic semiconductors, such as NFAs, is essential for a deeper understanding of the doping process, doping optimization, and improvement of the performance of OPVs.

p‐Doping of donor materials can have a beneficial impact on solar cell performance [25, 26]. Molecular p‐doping of poly(4′,3″‐dihexyl‐2,2′:5′,2″:5″,2″‐quaterfuran) (PF) with different concentrations of 2,3,5,6‐tetrafluoro‐7,7,8,8‐tetracyanoquinodimethane (F4TCNQ) in a bilayer structure with the acceptor C_60_ has been shown to lead to an increase in conductivity at doping ratios of 1%–2%, improving *J_sc_* without changing the photovoltaic band gap *E_pvg_* [25]. However, further increase of the doping concentration beyond 10%, while increasing *V_oc_* and *E_pvg_*, deteriorates the film morphology, negatively affecting the *J_sc_* and conductivity. Li et al. recently provided another example of OPV doping, demonstrating the effect of the 5,6,12,12a,13,18,18a,19‐octahydro‐5,6‐dimethyl‐ 13,18[1′,2′]‐benzenobisbenzimidazo [1,2‐b:2′,1′‐d]benzo[i][2.5]benzodiazo‐cine potassium triflate adduct (DMBI‐BDZC) on PM6:Y6 blends [26]. Their study showed that small dopant concentrations could improve charge transport and enhance bulk‐heterojunction (BHJ) OSC performance.

The use of organic salts like trityl tetrakis(pentafluorophenyl) borate (TrTPFB) as p‐dopants in conjugated polymers has also been studied to improve charge transport properties in organic semiconductors [27]. A study by Guo et al. proposed the p‐doping mechanism of TrTPFB in poly(3‐hexylthiophene‐2,5‐diyl) (P3HT) with high doping efficiency (80% to 100%) [27]. The trityl cation (Ph_3_C^+^) undergoes electrophilic attack on an electron‐rich thiophene of P3HT, creating a tritylated thiophenium ion (Wheland intermediate). This leads to an electron transfer from a neighbouring P3HT chain that forms two radical species: a neutral radical and a radical cation. Thus, the organic salt TrTPFB can be used as a p‐dopant to enhance the charge transport and conductivity of organic semiconductor materials by passivating trap states or stimulating the generation of additional free charge carriers [21, 28].

Despite these advancements, the mentioned studies on p‐doping primarily focused on donor materials or donor‐acceptor blends and not neat organic acceptors, such as modern NFAs, leaving this area unexplored. Studying these doping effects in BHJ devices does not provide a complete picture of the doping impact on the individual materials. Understanding p‐doping in NFAs only, such as Y6, is important for optimizing charge transport, tuning energy levels, and enhancing the performance of OPV devices. Moreover, controlling the doping of NFAs could enable the development of ‘bilayer’ single‐component p‐n junction OPV devices, offering a simplified device architecture with improved charge separation and transport. Studying p‐doping of NFAs, particularly on single‐component devices, could provide new insights into dopant‐host interactions and lead to more efficient strategies for improving the stability and efficiency of BHJ and bilayer OSCs.

In this work, we studied the doping effect on one of the most widely used NFAs, Y6, with TrTPFB as a p‐dopant [27, 29]. Controllable doping has been shown to passivate some of the trap states and decrease charge losses, leading to higher short‐circuit current and efficiency [18, 30]. To study the doping effect on a neat acceptor material, we fabricated a series of single‐component OSCs based on Y6 with different concentrations of the dopant. The results indicate that the addition of TrTPFB in Y6 can potentially reduce trap‐assisted recombination and influence the complex recombination mechanism, and thus improve charge transport and current density. The experiments showed that the power conversion efficiencies (PCE) of OPVs increase with the increase of the dopant concentration, starting at 0.03% for neat Y6 and reaching a maximum of 0.52% at 7.3 mol% of TrTPFB in Y6 devices. This study deepens the understanding of the doping process in the NFA Y6, providing valuable insights that contribute to a clearer comprehension of the doping mechanism in BHJ and bilayer systems that will support the development of advanced, efficient solar cells.

## 2 Results and Discussion

### 2.1 Doping Mechanism of TrTPFB

**FIGURE 1:**
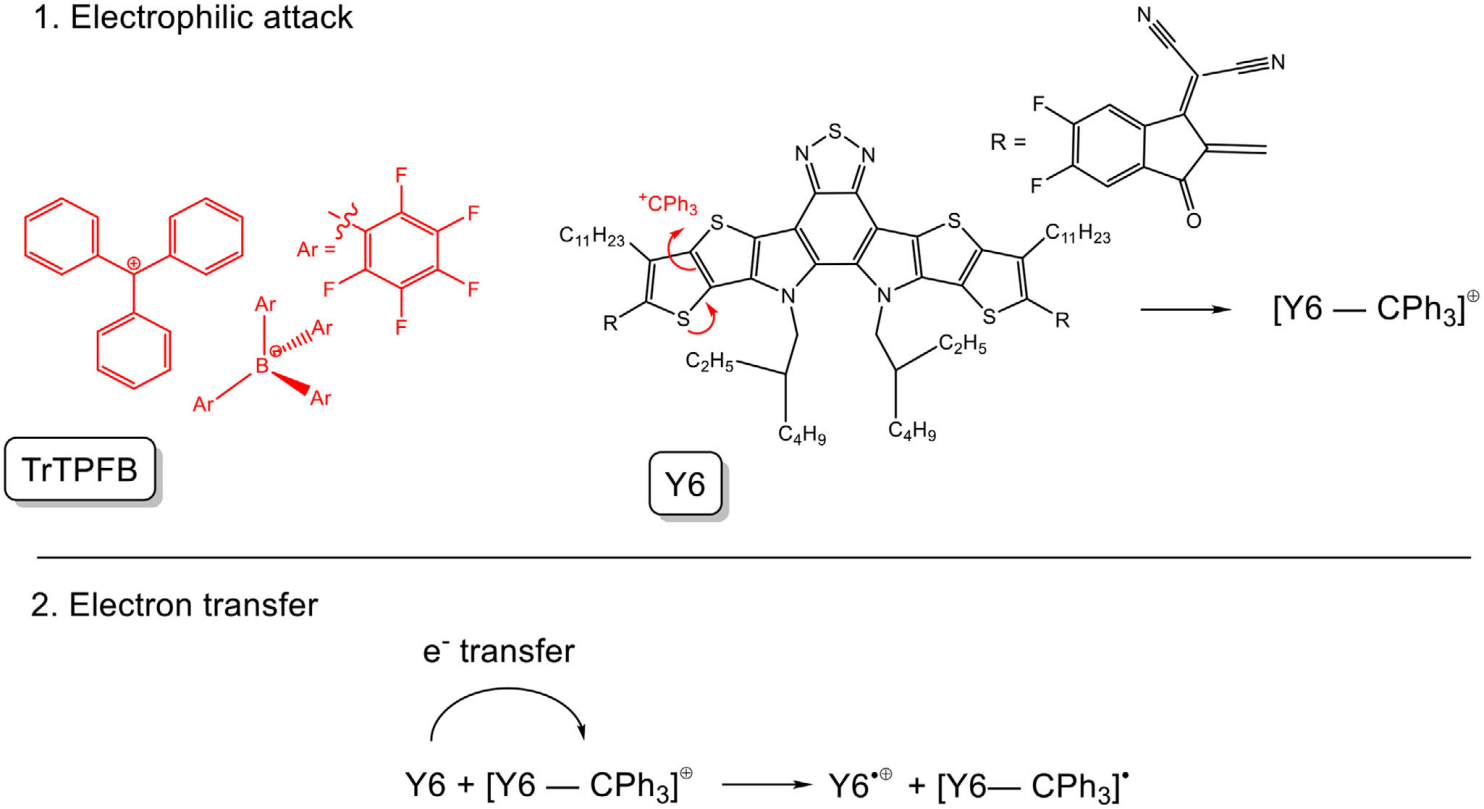
Proposed mechanism of p‐doping of Y6 with TrTPFB [27]. Step 1: electrophilic attack of trityl cation by one of the thiophene groups in Y6 leads to a positively charged Wheland intermediate; step 2: electron transfer from Y6 to the Wheland intermediate forms a neutral radical and positively charged Y6 molecule.

As suggested by Guo et al. the doping mechanism of TrTPFB can be interpreted for p‐doping of Y6. We hypothesize that the electrophilic attack of the trityl cation by one of the thiophene groups in Y6 leads to a positively charged Wheland intermediate [27]. Electron transfer from a neighbouring Y6 molecule then leaves a neutral radical and a positively charged Y6 molecule (Figure 1). The positively charged Y6 molecules formed from this process can potentially quench intrinsically n‐doped Y6 molecules and other negatively charged defects, which otherwise act as hole traps. Moreover, Guo's study clarifies that although the TrTPFB's anion component isn't directly involved in the doping reaction, it significantly influences the doping efficiency [27].

It is worth mentioning that the doping efficiency of electron acceptors, such as Y6, is expected to be low, compared to the doping of donor materials. For example, Guo and others claim 80% doping efficiency in P3HT with TrTPFB [27]. Electron acceptors have low‐lying HOMO levels, meaning it is more difficult to remove their electrons. Therefore, the doping reaction of Y6 is expected to be less efficient compared to donor materials.

### 2.2 Impact of TrTPFB Addition on the Optical Absorption and Photoluminescence of Y6

**FIGURE 2:**
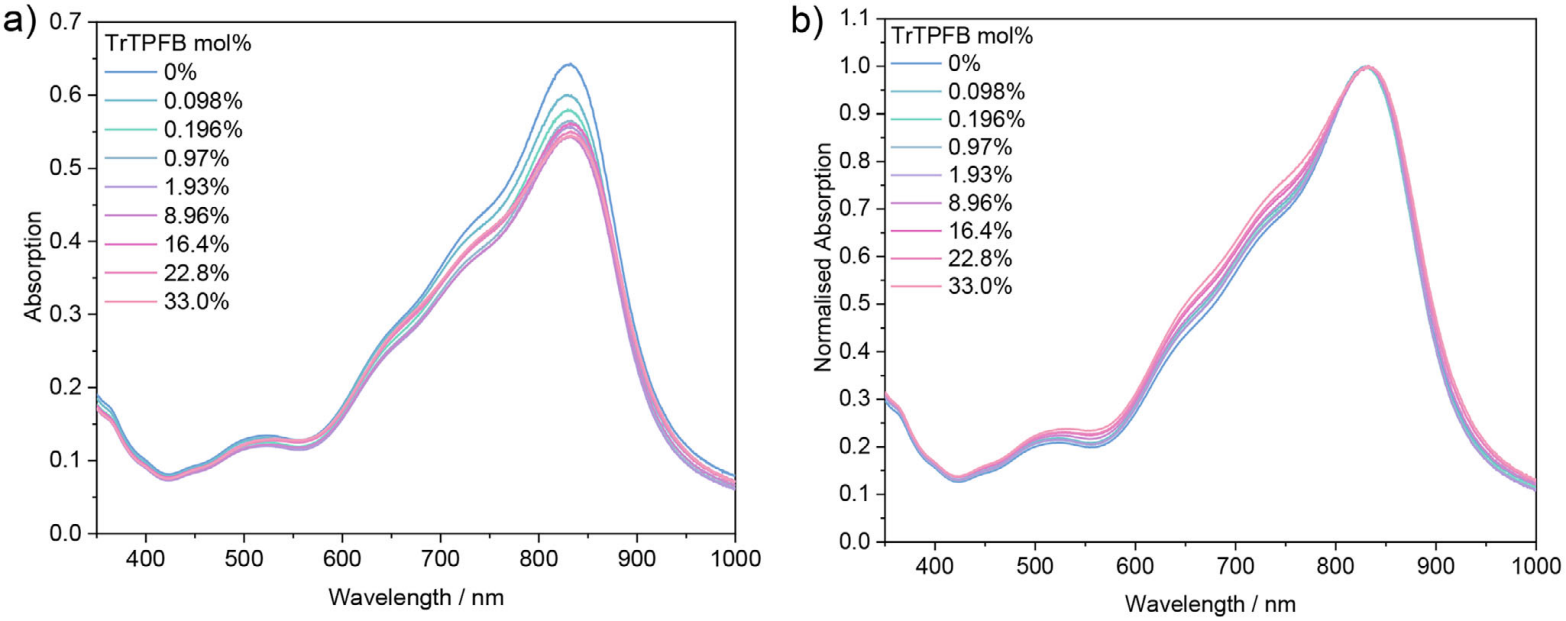
(a) UV–Vis absorption and (b) normalized absorption spectra of Y6 thin films with different concentrations of TrTPFB.

To study the effect of doping on the optical absorption and photoluminescence (PL) properties of Y6, we fabricated thin films (preparation details in SI Section 1). The UV–vis absorption spectra of p‐doped Y6 with TrTPFB thin‐films show that as the doping concentration increases, the absorption peak at ∼800 nm gradually decreases in intensity and saturates at ∼0.97 mol% (Figure 2). The doping efficiency of Y6 cannot be directly obtained from the decrease in absorption intensity, as this reduction may not be only due to doping, as it might exhibit light scattering, slight variations in film thickness and other effects. At the same time, we observe a slight increase of the shoulder between 600–800 nm when comparing normalised absorption spectra (Figure 2b).

**FIGURE 3:**
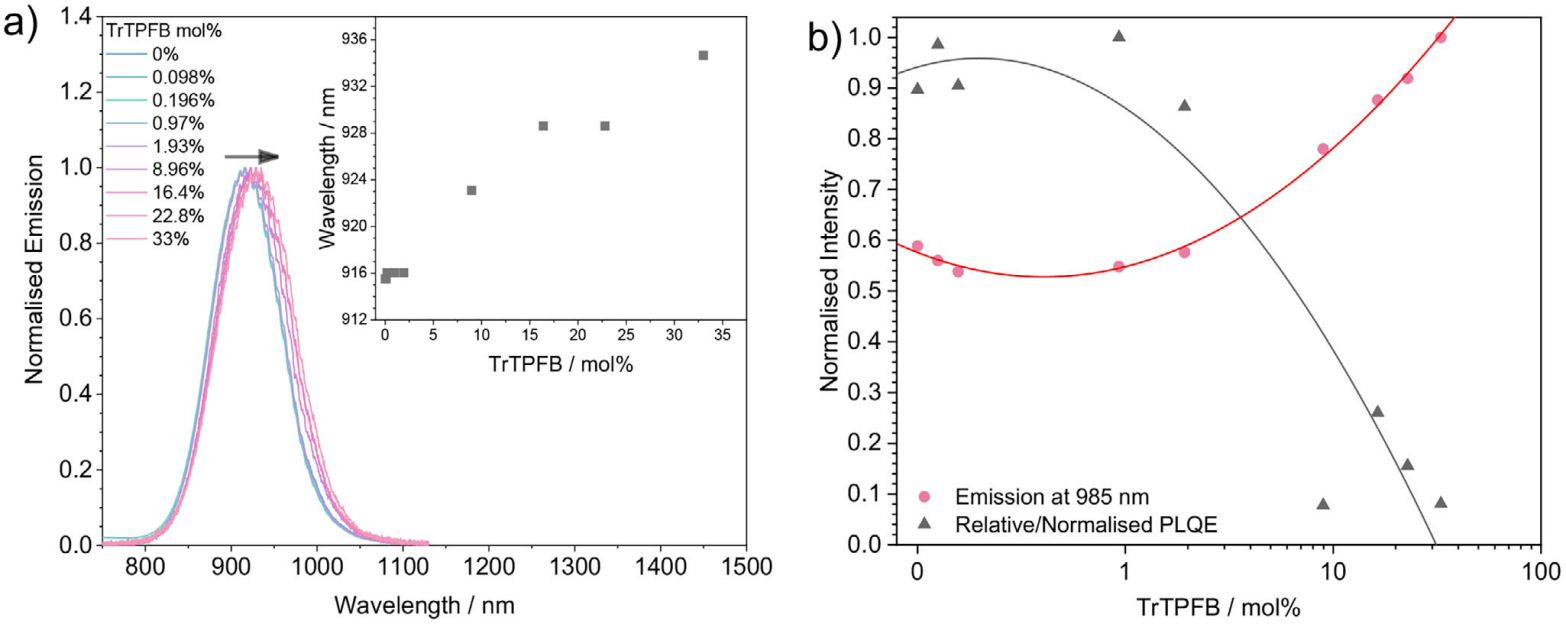
(a) Normalized emission spectra showing a redshift with increasing dopant concentration and (b) emission at 985 nm, and relative/normalised PLQE of Y6 thin films with different concentrations of TrTPFB.

The PL spectra of p‐doped Y6 films with varying concentrations of TrTPFB were measured under 520 nm excitation. As shown in the inset of Figure 3a, the PL peak exhibits a linear red‐shift with increasing dopant concentration, shifting from 915 nm for pristine Y6 to 935 nm at 33 mol% TrTPFB. Figure 3b presents the variation in relative PLQE intensity and sub‐gap emission (985 nm) as a function of dopant concentration. The relative or normalised PLQE was obtained by dividing the integrated PL by the fraction of absorbed photons at 520 nm and then normalising to the maximum value across all films, using the following equation:

$$PLQE=\frac{I_{PL}}{I_{Abs}}=\frac{I_{PL}}{I_{0}\cdot \left(1-10^{-Abs_{520\;\mathrm{nm}}}\right)}$$

where *I_PL_* number of emitted photons from integrated PL (Figure S7), *I_Abs_* is the number of total absorbed photons from the 520 nm laser excitation, and *I_0_* is the laser light intensity at 520 nm. To obtain the relative or normalised PLQE values, we can take *I_PL_* in detector counts and calculate *I_Abs_* from the absorption values at 520 nm (Abs_520 nm_) and the laser intensity *I_0_* of 2 µW (the same for all samples). These values are proportional to the corresponding quantities expressed in photon counts, which means that the resulting ratio *I_PL_*/*I_ABS_* is proportional to the PLQE. The PL sub‐gap features in normalised spectra are of particular interest, as they are associated with trap states within the bandgap. Initially, emission decreases with the introduction of the dopant, indicating trap filling [31]. At higher concentrations, however, these features increase again, suggesting the formation of additional trap states [22]. The corresponding rise and subsequent decline in relative or normalized PLQE further support this interpretation, pointing to enhanced radiative recombination as traps are filled, followed by quenching due to trap overfilling. Collectively, these results demonstrate a trap‐filling effect and the existence of an optimal doping concentration, beyond which parasitic trap states dominate the sub‐gap optical response.

### 2.3 Influence of TrTPFB Addition on Single‐Component Solar Cells

**FIGURE 4:**
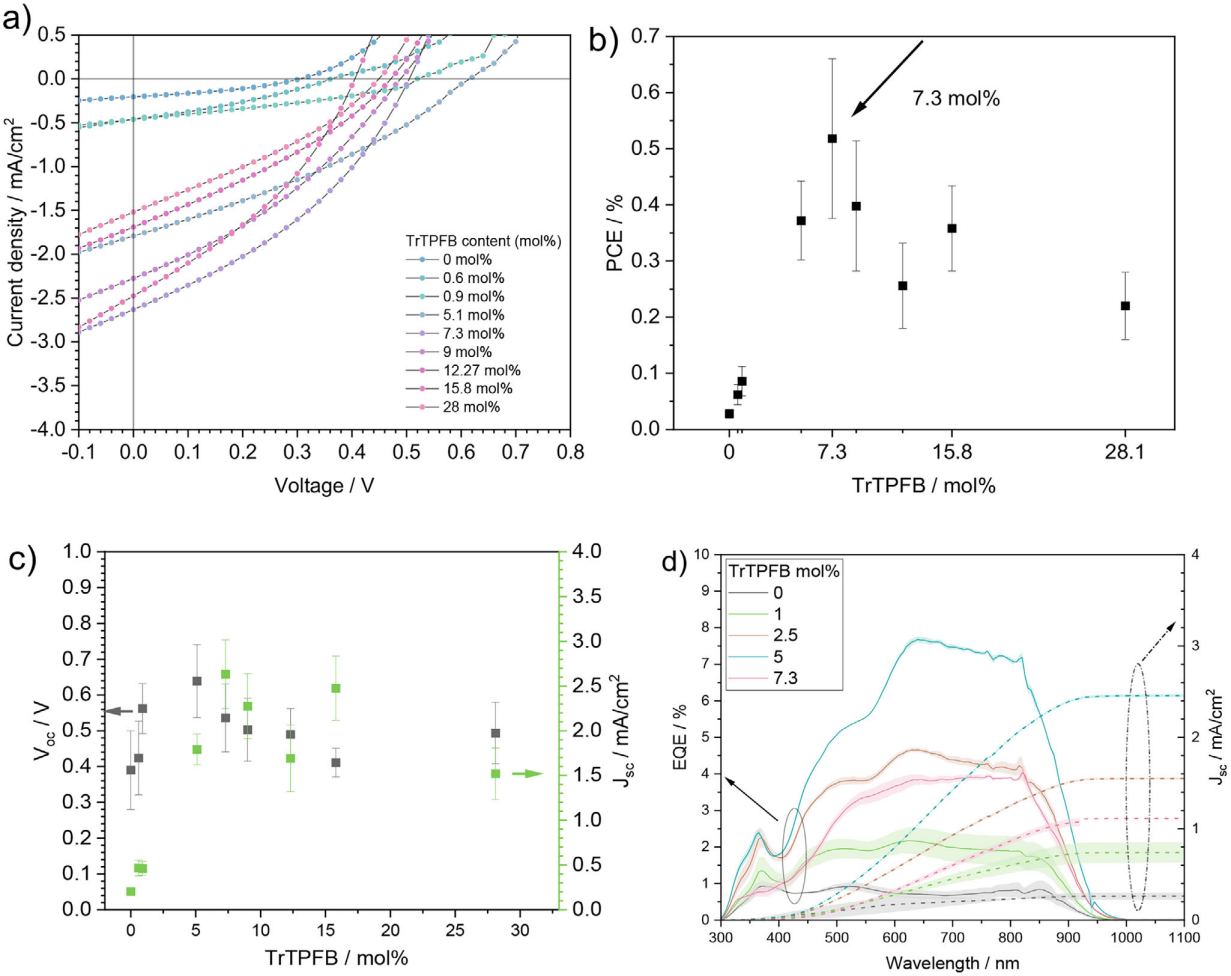
(a) *J–V* curves of OSC based on Y6 with different concentrations of TrTPFB; (b) PCE versus concentration of TrTPFB in mol %; (c) *V_oc_* and *J_sc_* of OSC based on p‐doped Y6; (d) EQE profiles of neat Y6 and doped Y6 OSCs with TrTPFB.

To study the photovoltaic properties of p‐doped Y6 films, inverted architecture OSCs were fabricated as described in the device preparation section, with the structure ITO/ZnO/Y6/MoO_3_/Ag (Section S1). A series of Y6 films for OSCs was prepared with different concentrations of TrTPFB (Table S1). The amount of TrTPFB doped in Y6 strongly affects optoelectronic properties: the PCE of neat Y6 OSC (0.03% ± 0.01%) is significantly lower than that of the highest efficiency doped cells with 7.3 mol% of TrTPFB (0.52% ± 0.14%) (Figure 4a,b).

As the TrTPFB content increases, the PCE of OCSs rises to 0.52% at 7.3 mol%, but then decreases to 0.22% ± 0.06% for 28.1 mol%. This dependence demonstrates that doping initially improves device efficiency but may eventually introduce additional charge/exciton traps or alter the film morphology (Figure S2a,b), decreasing the PCE of the devices. This correlates with the average *J_sc_* of the devices: neat Y6 OSCs have 0.2 mA/cm^2^, and OSCs with 7.3 mol% of TrTPFB have 2.6 mA/cm^2^ (Figure 4c). Nonetheless, the maximum achieved *V_oc_* is 0.64 V for 5 mol% of TrTPFB films, compared with 0.39 V for neat Y6 (Figure 4c).

A similar trend is observed for external quantum efficiency (EQE), which rises up to 5 mol% before declining (Figure 4d). In contrast, the *J–V* curve data indicate that the highest‐performing device contains 7.3 mol% of the dopant. The EQE measurements were conducted on devices fabricated using a different batch of Y6 from another supplier, likely resulting in a slight variation in trap density, which could explain the shift from 7.3 mol% to 5 mol% of the optimal concentration. Overall, despite the different optimal concentrations for OSCs for *J‐V* and EQE measurements, the key observation is the consistent trend with rising performance up to the optimal concentration for Y6 and then decreasing with excess dopants.

The best‐performing device, containing 7.3 mol% TrTPFB demonstrates a PCE of 0.67%, a fill factor of 37.2%, *V_oc_* of 0.61 V, and *J_sc_* of 2.92 mA/cm^2^ (Figure 4a). These correlations between *J_sc_*, *V_oc_*_,_ and dopant content likely represent the improvement of the trap‐state filling in the active layer within the inverted device architecture. Controlled addition of the dopant in Y6 significantly enhances device performance, with optimal properties achieved at 7.3 mol% TrTPFB.

### 2.4 Impact of TrTPFB Addition on Light Intensity‐Dependent Device Performance

**FIGURE 5:**
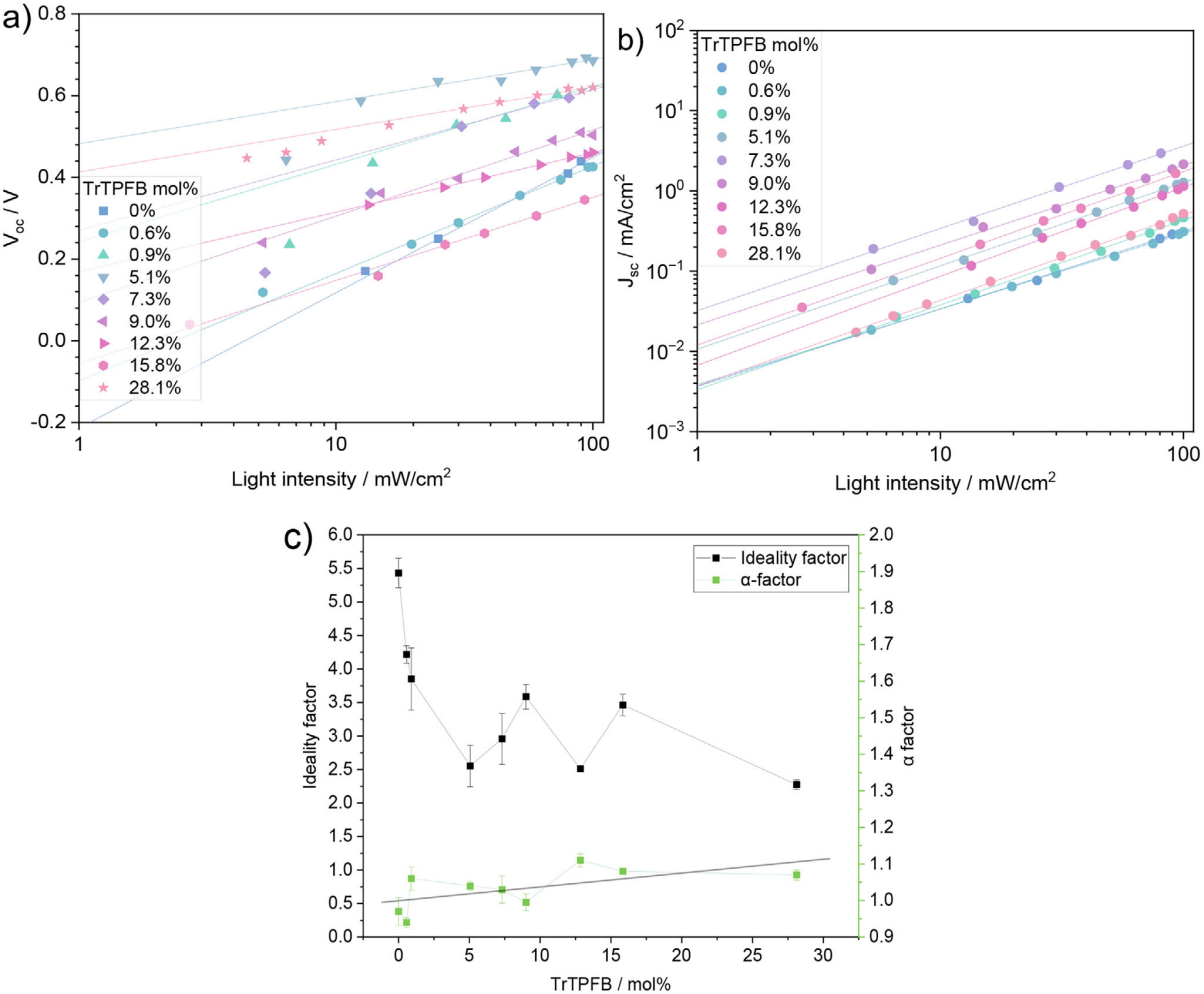
(a) *V*_oc_ versus light intensity, and (b) *J*_sc_ versus light intensity for Y6‐based OSC with different dopant concentrations, (c) ideality‐ and α‐factors.

The analysis of the light intensity dependence of *J–V* curves can demonstrate the recombination mechanisms operating in the films. Device measurements under different light intensities were conducted for the Y6‐based OSCs of each dopant concentration (Figure 5). Light intensity‐dependent *J–V* curves of the p‐doped devices are presented in Figure S3, where the *J*_sc_ and *V*_oc_ increase linearly with light intensity. The light‐dependent *V*_oc_ and *J*_sc_ of the best‐performing device at these concentrations are shown in Figure 5a,b, respectively.

The ideality factor *n_if_* can be calculated using the following equation:

$$n_{\mathrm{if}}=\frac{e}{\mathrm{kBT}}\frac{\mathrm{dVoc}}{d\left(\log\left(L\right)\right)}$$

where *e* is the elementary charge, *k* is the Boltzmann constant, *T* is the absolute temperature, and *L* is the light intensity. The ideality factor extracted from the light intensity‐dependent experiments decreased for p‐doped films in comparison with neat Y6, but was still quite high (*n_if_* > 2), indicating a complex recombination mechanism and interface‐related issues [32, 33, 34]. The ideality factor decreases from 5.4 down to a range 2.3–3.6 for devices with TrTPFB content of over ∼5 mol%. Moreover, a high ideality factor >2 might indicate decreased energy disorder in Y6 films, which can potentially be a sign of trap passivation [35]. At the same time, the linear slope of *J*_sc_ vs. light intensity shows the increase of the α‐factor (α = *d*log (*J_sc_*)/*d*log (*L*)) to value greater than 1, which can potentially represent increased charge extraction efficiency with an increase in the dopant concentration [32, 34]. Moreover, the α‐factor exceeds 1 for devices with the dopant concentration over 0.9 mol%, which might be a signature of trap‐filling behavior. This suggests that TrTPFB addition in Y6 can potentially reduce complex recombination mechanisms, and enhance the interface related issues, potentially filling the trap states in Y6, leading to improved short‐circuit current and open‐circuit voltage, and overall device performance.

### 2.5 Tuning the Charge Transport Properties via TrTPFB Addition in Y6

**FIGURE 6:**
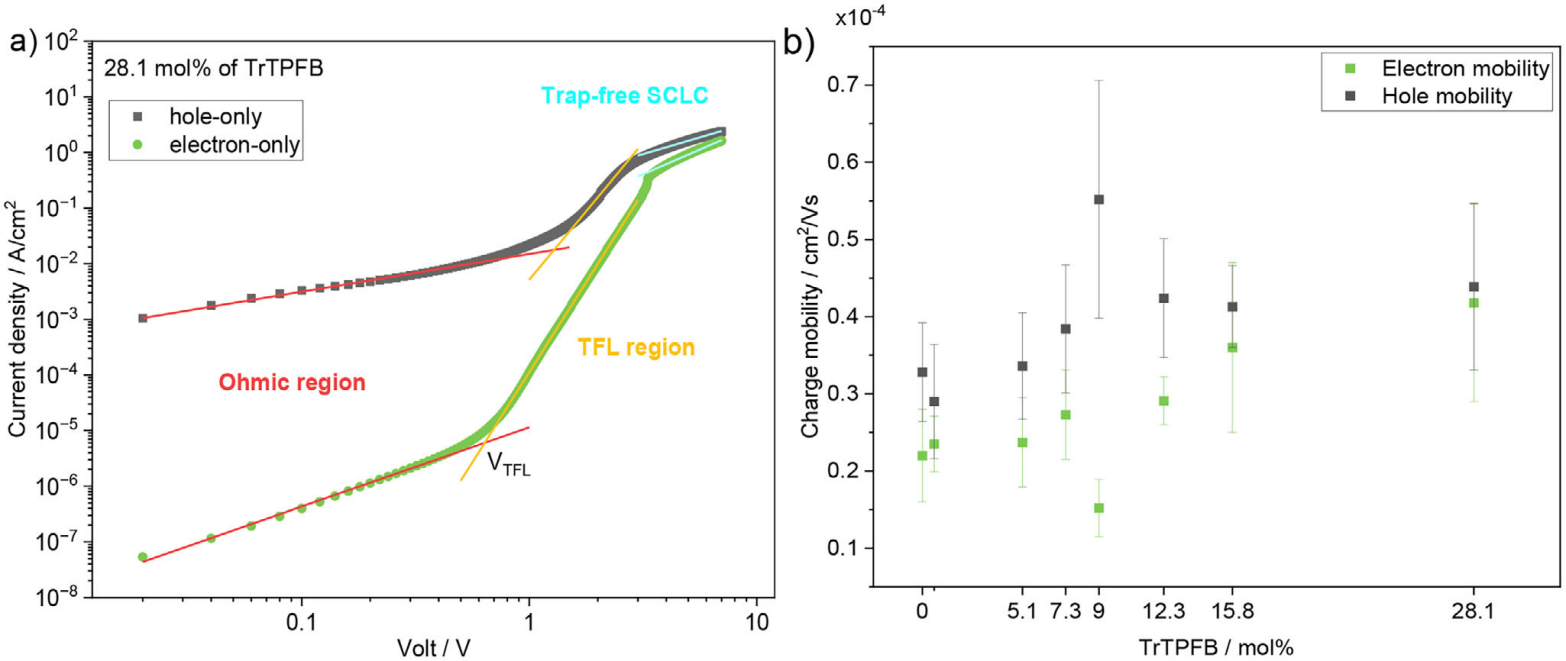
(a) *J–V* curves for hole and electron‐only devices; (b) Hole and electron mobilities for p‐doped with TrTPFB Y6 films.

To find out how the TrTPFB impacts the charge transport and charge traps, we fabricated hole and electron‐only devices, using the space‐charge limited current method (SCLC) [22, 36, 37]. *J–V* curves are comprised of three regions: the ohmic region, trap‐filled limit (TFL) region, and trap‐free SCLC for hole and electron‐only devices (Figure 6a) [38]. An ohmic region can be characterized by the slope of 1 in the *log(J)* vs *log(V)* dependence. Ideal ohmic SCLC devices should demonstrate α = *d*log (*J*)/*d*log (*V*) equal 1, or close to 1 in near ohmic SCLC devices. As demonstrated in Figure S6, the α values for most of the samples are 1 or ∼1. The deviation from 1 for some samples could potentially be due to an injection barrier, defects, or fabrication issues. Further, we assume that the sample's deviation from 1 is low and negligible, and the fabricated SCLC devices are ohmic.

The hole and electron mobilities were estimated using the following equation:

$$J_{\mathrm{SCLC}}=\frac{9}{8}\frac{\varepsilon_{0}\varepsilon_{r}\mu {\left(V_{\mathrm{app}}-V_{\mathrm{bi}}\right)}^{2}}{d^{3}},$$

where J_SCLC_ is the current density in the SCLC region, *V*_app_ is the applied voltage, *V*_bi_ is the built‐in voltage, *µ* is charge mobility, d is the film thickness, ε_r_ is the relative dielectric constant, and ε_0_ is the constant of permittivity [38]. With increasing dopant concentration, hole mobility rises from 0.33·10^−4^ cm^2^/Vs in neat Y6 films to the maximum of 0.55·10^−4^ cm^2^/Vs at 9 mol%. Electron mobility in the films gradually increases from 0.22 to 0.42 cm^2^/Vs (Figure 6b).

**FIGURE 7:**
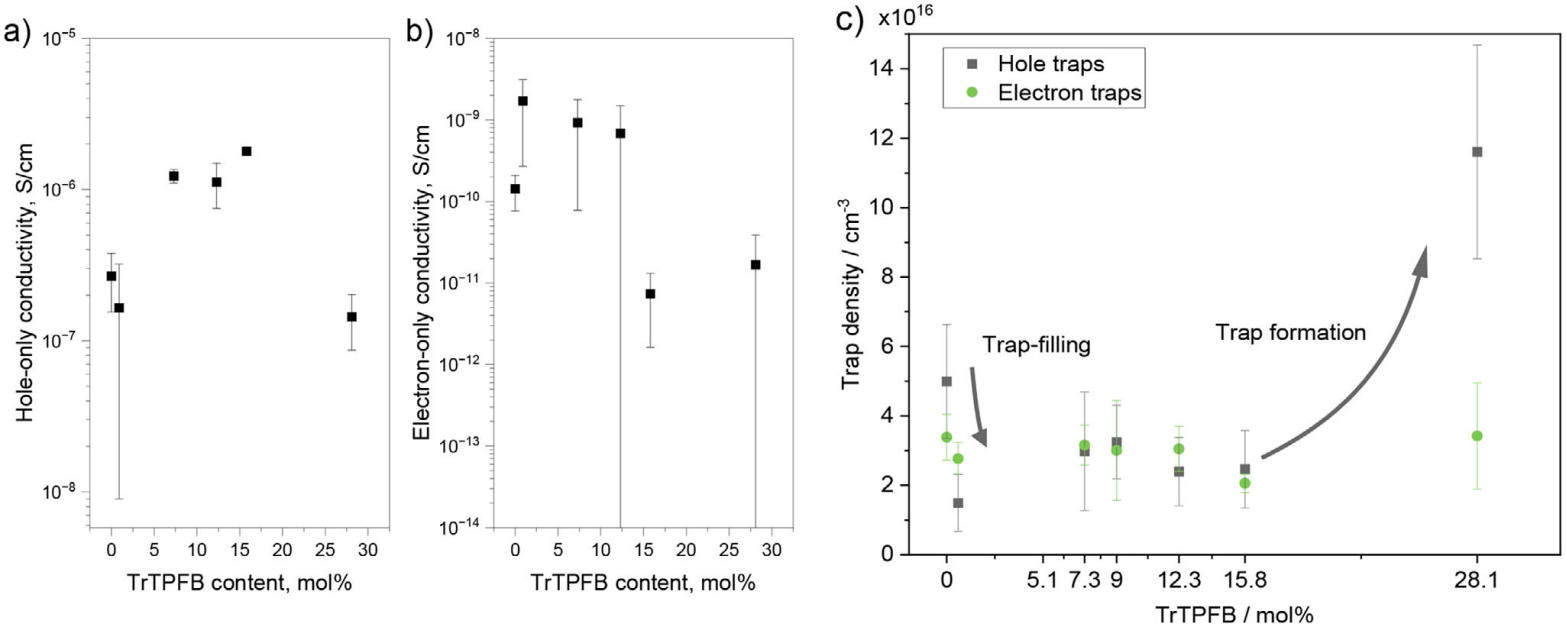
(a) Conductivity of the p‐doped Y6 in hole‐ and (b) electron‐only devices; (c) Hole and electron trap density in the p‐doped Y6.

The conductivity of hole‐ and electron‐only devices was measured in the ohmic regime (Figure 7a,b). The experiments demonstrate an increase in hole conductivity, followed by a decrease at higher doping concentrations. The electron conductivity increases at lower concentrations and then gradually decreases. However, the overall effect of the dopant on the electron conductivity is negligible.

To estimate the influence of the dopant on hole and electron trap densities in Y6, we calculated these values using the following equation:

$$n_{\mathrm{trap}}=\frac{2\varepsilon_{0}\varepsilon_{r}V_{\mathrm{TFL}}}{ed^{2}},$$

where *V*_TFL_ is trap‐filled limit voltage [39]. The estimated trap density shows a reduction in hole traps at lower dopant concentrations, which then remains steady with further increases in dopant concentration, up until 28 mol%, where the films exhibit a rise in hole trap density (Figure 7c). This may suggest the formation of additional hole traps at higher doping levels [22]. Conductivity and trap density measurements reveal a reduction in hole traps and a peak in the hole conductivity at moderate doping levels.

Overall, the systematic doping of Y6 with TrTPFB reduces the trap density, leading to the improvement of conductivity, the enhancement of hole and electron charge transport, and ultimately to improved short‐circuit current in the solar cells. This illustrates how optimizing charge transport and trap reduction with controlled TrTPFB addition can be an essential part of performance maximization in OSCs.

## 3 Discussion

Controlled TrTPFB addition in Y6 thin films significantly enhances the optoelectronic properties of the single‐component solar cells. The introduction of the dopant improves the charge transport by possible passivation of trap states, suppressing complex recombination mechanism, improving interface‐related issues, and increasing both hole and electron charge mobilities. These changes significantly improve current density and performance in single‐component solar cells at the optimal doping concentration of 7.3 mol% compared to neat Y6 devices.

As observed in light‐intensity dependent experiments, the reason for the current density improvement might be in the improvement of the charge extraction due to the interface alignment and reduction of the energy disorder [40, 41]. The decrease in ideality factor with increasing TrTPFB content in Y6 indicates a reduction in interface‐related recombination losses, suggesting that the interfaces between the transport layers (ZnO and MoO_3_) and Y6 are becoming more Ohmic and allowing easier charge extraction.

It was observed that the optimal doping concentration differs between film measurements (Figure 3b) and OSC device parameters. The observed trap density in SCLC experiments is at ∼10^16^ cm^−3^, which corresponds to 1 trap per ∼10^5^ Y6 molecules (assuming a density of ∼1.2 g/cm^3^, which corresponds to a ∼10^21^ cm^−3^ desnity of Y6 molecules). The overall changes in spectroscopic properties are therefore expected to be subtle. However, these changes only show the presence of the trap‐filling effect rather than a quantitative measure of its impact on device performance. On the other hand, the OSC devices include transport layers, and efficient charge extraction plays a dominant role. The introduction of TrTPFB can also modify the interfacial alignment with the transport layers and reduce the energetic disorder, leading to different optimal doping concentrations for films and for devices. Moreover, the trap‐filling effect in the optical experiments appears relatively small compared to the overall device improvement. We believe that the position of the minima for the parabolic curves in Figure 3b can only indicate the presence of the trap‐filling effect rather than a quantitative measure of its impact on device performance. The optical measurements only show that there is a potential trap passivation, without accounting for the effect on the charge transport/electrical characteristics of the OSCs.

It is important to note that the optical measurements (Figure 3b) provide the most direct evidence of trap‐filling behavior. The systematic changes in PLQE and sub‐gap emission are difficult to explain without trap filling, which is consistent with a p‐doping mechanism involving trap passivation. Furthermore, the different optimal doping concentrations observed in films and devices represent an intriguing result, indicating that p‐doping influences both bulk and interfacial processes.

An increase in hole and electron conductivity was observed in the hole‐only devices. Moreover, at low dopant concentration below 12.3 mol%, a change in electron conductivity in electron‐only devices was detected, which may happen due to the reduction of the energy gap between the transport layers (ZnO and LiF/Ag) and Y6 [42]. At high dopant concentration, this effect is likely due to Coulomb scattering, and the electron conductivity decreases (Figure 7a,b) [43, 44]. Moreover, the low overall electron conductivity may be due to the alternation of the top electrode (Ag) work function by LiF, which may not be ideally matched with the Y6's LUMO level. At the same time, the dopant shifts the Fermi level even deeper, extending the barrier at the Y6/LiF/Ag interface, and limiting electron injection [45, 46]. This possibly explains why neat Y6 demonstrates lower electron mobility compared to hole mobility, and electron conductivity compared to hole conductivity, which may be masked due to the high contact resistance of the electron‐only device.

Despite the potential existence of multiple molecular species (Y6, doped Y6, and Y6 – neutral radical), it seems like a BHJ‐like film is not forming in the Y6/Y6 – neutral radical system [47]. Typically, BHJ formation is accompanied by significant morphological changes and segregation [48]. The numerical calculations of HOMO/LUMO levels revealed a possible charge transfer from Y6‐neutral radical to Y6, albeit at negligible driving force (Figure S4). We do not observe evidence of this electron transfer by transient absorption measurements (Figure S5). Transient absorption experiments showed no significant differences in lifetimes between neat Y6 films and doped Y6 (5 mol%), which would be higher for the BHJ‐like system (Section S6). Thus, the experiments demonstrated that a BHJ‐like system is unlikely to have formed as a result of the doping process.

The excess dopants and counter‐anions are likely not participating in any charge transport, but they can potentially affect the morphology of the films, such as surface roughness and overall film quality. The observed root mean square (RMS) surface roughness obtained by atomic force microscopy (AFM) demonstrates a change from 1.4 to 2.4 nm while remaining relatively low and smooth (Figure S2). However, no significant morphological changes or phase separation are observed in the experiments (Figure S2). Figure 2 shows that the UV–Vis shape does not change significantly with the dopant introduction, which suggests that the morphology changes are insignificant [49]. Moreover, excess dopants can create more traps, as Y6 can have only a limited amount of charge traps.

Despite the undoubted improvements in Y6‐based OPV cell efficiency, the explanation that the addition of TrTPFB leads to p‐doping and passivates hole traps would benefit from additional direct evidence, which would strengthen the case for p‐doping. Currently, we have demonstrated several signatures consistent with a p‐doping effect, including trap filling observed in PL and PLQE, α‐factor >1 indicating trap filling and improved charge extraction, increase in hole conductivity and mobility, decrease in *n_trap_*, and improved OSC performance. The Mott‐Schottky analysis would help to prove the p‐doping effect, providing more convincing evidence. In this study, we can confirm only the signs of a p‐doping effect discussed earlier.

These results demonstrate the broad potential of p‐doping control as a strategy to enhance the performance of NFAs in organic solar cells by improving charge transport, potentially reducing trap‐assisted recombination, and increasing current density and device efficiency. Finally, the ability to control doping in organic solar cells provides valuable insights for developing the next generation of organic solar cells, such as single‐component and bilayer devices, bringing their performance to the next level.

## 4 Conclusion

Single‐component OSCs based on p‐doped Y6 with TrTPFB were fabricated to study how doping affects the optoelectronic properties. TrTPFB addition potentially passivates hole traps, leading to significant improvements in short‐circuit current, open‐circuit voltage, and overall organic solar cell performance. Systematic doping of Y6 with TrTPFB was found to enhance charge extraction, charge transport, and potentially reduce trap‐assisted recombination. The PCE of OSCs increases with the increase of the TrTPFB concentration, with the maximum average of 0.52% (0.67% max) at 7.3 mol%. These findings highlight the broader importance of controlled p‐doping as a strategy to maximize the performance of organic solar cells and offer valuable insights for advancing bilayer and bulk‐heterojunction device architectures alike.

## Author Contributions

**Nikita A. Shumilov**: investigation, writing – original draft, methodology, visualization, writing – review and editing, formal analysis, project administration, data curation. **Aditi Kumar**: investigation, methodology, writing – review and editing, formal analysis. **Justin M. Hodgkiss**: conceptualization, supervision, writing – review and editing, formal analysis, funding acquisition, validation. **Paul A. Hume**: conceptualization, supervision, writing – review and editing, formal analysis. **Michael B. Price**: conceptualization, supervision, writing – review and editing, formal analysis, validation. **Nathaniel J. L. K. Davis**: supervision, writing – review and editing, formal analysis, validation.

## Conflicts of Interest

The authors declare no conflicts of interest.

## Supporting information




**Supporting File**: smll74397‐sup‐0001‐SuppMat.docx.

## Data Availability

The data that support the findings of this study are available from the corresponding author upon reasonable request.
