# Supplementary material for: Controlling the Optoelectronic Properties of the Non‐Fullerene Acceptor Y6 via p‐Doping
Source: Small. 2026 Jul 8;22(48):e74397. doi: 10.1002/smll.74397 (PMC13509069; doi:10.1002/smll.74397)
Supplement: Supplementary file 1 [file SMLL-22-e74397-s001.docx]

**Supplementary Information for**

**Controlling the optoelectronic** **properties of the non-fullerene acceptor Y6 via p-doping**

*Nikita A. Shumilov^a-c^, Aditi Kumar^a-c^, Paul A. Hume^a-c^, Michael B. Price ^d *^, Nathaniel J. L. K. Davis^a-c *^, Justin M. Hodgkiss^a b *^*

^a^ School of Chemical and Physical Sciences, Victoria University of Wellington, Wellington 6140, New Zealand.

^b^ The MacDiarmid Institute for Advanced Materials and Nanotechnology, Wellington 6140, New Zealand.

^c^ The Dodd-Walls Centre for Photonic and Quantum Technologies, University of Otago, 12 Dunedin 9056, New Zealand.

^d^ School of Chemistry, University of Bristol, Bristol, BS8 1TS, United Kingdom.

**Corresponding Authors**

**Michael B. Price** *−* School of Chemistry, University of Bristol, Bristol, BS8 1TS, United Kingdom. Email: [michael.price@bristol.ac.uk](mailto:michael.price@bristol.ac.uk)

**Nathaniel J. L. K. Davis** *− School of Chemical and Physical Sciences, Victoria University of Wellington, Wellington 6012, New Zealand; MacDiarmid Institute for Advanced Materials and Nanotechnology, Wellington 6012, New Zealand; The Dodd-Walls Centre for Photonic and Quantum Technologies, Dunedin 9016, New Zealand;* Email: [nathaniel.davis@vuw.ac.nz](mailto:nathaniel.davis@vuw.ac.nz)

**Justin M. Hodgkiss** *− School of Chemical and Physical Sciences, Victoria University of Wellington, Wellington 6012, New Zealand; MacDiarmid Institute for Advanced Materials and Nanotechnology, Wellington 6012, New Zealand;* Email: [justin.hodgkiss@vuw.ac.nz](mailto:justin.hodgkiss@vuw.ac.nz)

# Section 1 – Experimental methods

*Materials:*

Y6 was purchased from Solarmer and Organtec.

Trityltetra(pentafluorophenyl)borate (TrTPFB) was purchased from Stream.

Chloroform (anhydrous, 99%), and Lithium fluoride were purchased from Sigma Aldrich.

Ethanol (anhydrous, 99%), acetone (analytical reagent grade, 99.8%), and isopropanol (analytical reagent grade, 99.8%) were purchased from Fisher Chemicals.

Poly(2,3-dihydrothieno-1,4-dioxin)-poly(styrenesulfonate) (PEDOT:PSS) in water (1:2.5 w/w) and prepatterned indium tin oxide (ITO) glass substrates (15 Ohm) was purchased from Ossila.

Silver (99.99%) and MoO_3_ were purchased from Angstrom.

*Organic solar cells and sample preparation:*

The ITO substrates were sonicated in ethanol, acetone, and isopropanol for 5 minutes each, and dried with a dry air gun. A ZnO layer was used as the electron transport layer (ETL) in the solar cells because the conductive energy level of ZnO well fits with the Y6 LUMO level, and it increases the stability of devices.^1­^ A solution of diethylzinc in hexane (1M) was diluted in anhydrous tetrahydrofuran (1:3) in a nitrogen-filled glovebox. The solution was taken outside the glovebox to an ambient environment where 50 µl was spin-coated at 5000 r.p.m. for 30s on top of the cleaned ITO substrates. The substrates were allowed to rest for 10 minutes in an ambient environment and then annealed at 135°C for 10 minutes.

For preparing the active layer (AL) for a device or for a glass substrate, Y6 was dissolved in chloroform at 28 mg/mL and stirred for 2h at 65°C in a nitrogen-filled glovebox. Separately, TrTPFB was dissolved in chloroform at 5 mg/mL for 2h at 65°C. These solutions were mixed with a proportion to achieve a certain mol percentage of TrTPFB in the final solution (Table S1) and stirred together for 20 min. Additional chloroform was added to make the final concentration of Y6 14 mg/mL. The solution was filtered with a 0.22 µl filter and spin-coated at 3000 r.p.m. for 60s onto either a glass substrate or a ZnO treated ITO slide. The thickness of all Y6 films is ~85 nm.

**Table S1.** Experiments for p-doping of Y6 with TrTPFB

| TrTPFB  %mol | Y6, ml  (1451.93 g/mol) | TrTPFB, ml  (922.37 g/mol) | Additional ChCl_3­_, ml | Final TrTPFB mg/mL |
| --- | --- | --- | --- | --- |
| 0 | 0.7 | 0 | 0 | 0 |
| 0.56 | 1 | 0.02 | 0.98 | 0.05 |
| 0.90 | 0.8 | 0.032 | 0.768 | 0.089 |
| 5.06 | 1 | 0.18 | 0.82 | 0.45 |
| 7.31 | 0.9 | 0.26 | 0.74 | 0.68 |
| 9.0 | 1 | 0.32 | 0.68 | 0.8 |
| 12.27 | 0.7 | 0.28 | 0.42 | 1.2 |
| 15.84 | 0.7 | 0.42 | 0.28 | 1.55 |
| 28.11 | 0.7 | 0.7 | 0 | 2.5 |

The last step of solar cell fabrication is the evaporation of both the hole transport layer (HTL) and cathode. The substrates were moved to a glovebox-housed thermal evaporator (Angstrom) with a shadow mask for the samples which allows for 2x3 mm for each electrode, where 8nm of molybdenum trioxide (MoO­_3_) and then 100nm of silver (Ag) were thermally evaporated on top of the active layer. The final solar cell device architecture for these experiments is ITO/ZnO/AL/MoO_3_/Ag (Figure S1).

**Figure S1.** Inverted solar cell device architecture.

The current-voltage experiments were conducted in the ambient environment under AM1.5G illumination, 100mW/cm2, using Enlitech 3A Solar Simulator SS-R100B and Keithley 2401 SourceMeter. The light dependent measurements were conducted using neutral density filters and silicon solar cell calibration.

*External quantum efficiency measurements:*

External quantum efficiency (EQE) measurements were carried using an Enlitech QE-R instrument.

*Charge Transport Measurements, Space Charge Limited Current:*

To study charge transport characteristics, we used the space charge limited current method (SCLC). We fabricated 2 different types of devices with the architectures ITO/ZnO/AL/LiF/Ag for electron-only and ITO/PEDOT:PSS/AL/MoO_3_/Ag for hole-only devices. PEDOT:PSS films were spin-coated on top of pre-cleaned ITO substrates in the ambient environment at 5000 r.p.m. for 30s with a 150μL solution volume. Then the substrates were allowed to rest for 10 minutes in an ambient environment and then annealed at 135°C for 10 minutes. LiF and MoO_3_ films were thermally evaporated on top of an active layer (8 nm) (as described above).

The current-voltage experiments were conducted in the ambient environment Keithley 2401 SourceMeter.

*Photoluminescence Measurements:*

Photoluminescence (PL) measurements were carried out using a homebuilt setup and 520 nm continuous wave laser (520 nm, 2 µW, Ø5.6 mm Thorlabs L520P50). The diode temperature was controlled by a benchtop temperature 10 controller (Thorlabs TED200C). Kymera 328i Andor Spectrograph with a silicon detector (iDus 420 CCD) and a grating with 800 nm central wavelength was used to record the measurements. Intensity calibration of the data was done using a Stellar Net calibration lamp (1041993).

*Absorptance Measurements:*

Absorption spectra were measured using a Varian Cary 50 Bio UV-Vis spectrophotometer in range 300 to 1100 nm.

*Atomic force microscopy:*

The atomic force microscopy (AFM) measurements were conducted using Nanosurf, NaioAFM.

*Transient absorption spectroscopy:*

Transient absorption measurements were performed using a Yb-based fiber laser amplifier (Tangerine SP, Amplitude) operating at 1030 nm with a pulse duration of <120 fs and a repetition rate of 3 kHz. The fundamental output was split to generate both pump and probe beams.

For pump excitation, the 1030 nm fundamental was frequency-doubled in a BBO crystal to produce 515 nm pulses. The pump was directed through a motorized delay stage for precise temporal control relative to the probe and modulated by an optical chopper to alternate excited and unexcited sample conditions. The pump polarization was set to the magic angle (54.7°) with respect to the probe to suppress orientational effects. The pump beam was then focused onto the sample with a spot size (~94 µm 1/e^2^) larger than that of the probe (~335 µm 1/e^2^) to ensure uniform excitation within the probed region.

For probe generation, a portion of the 1030 nm fundamental was focused into a YAG crystal to generate a broadband white-light continuum. The probe was spatially overlapped with the pump at the sample position and re-collimated after transmission using an off-axis parabolic mirror. The transmitted probe was dispersed by a custom-built spectrometer and detected with a high-speed CMOS linescan camera (Glaz Linescan II, Synertronic, equipped with a Hamamatsu S11639-01 sensor). All measurements were performed on thin-film samples maintained under active vacuum in a custom-designed chamber.

*Density functional theory calculations:*

Density functional theory (DFT) calculations were performed using the long-range corrected CAM-B3LYP exchange–correlation functional in combination with the 6–31+G(d,p) basis set.^3^ Geometry optimisations were performed in vacuum with alkyl chains truncated to ethyl. Optimised geometries were confirmed by calculation of the associated vibrational modes, which revealed no imaginary frequencies in all cases. Linear response TD-DFT calculations for the excited states were performed within the Tamm–Dankoff approximation (TDA), with dielectric stabilisation treated using a polarisable continuum model with a static dielectric constant set to 3.8. For the excited state calculations, the exchange–correlation functional was range-tuned according to the established non-empirical procedure, resulting in an optimal range-separation parameter of 0.015 Bohr^-1^.^4–7^ All quantum chemical calculations were performed using Gaussian 16, Rev. C.01.^8^

**Section 2 –** **Atomic force microscopy**

Figure S2 shows AFM topographic images of the p-doped Y6 films. The introduction of TrTPFB insignificantly affects the film’s surface. The root mean square (RMS) roughness measured on AFM shows approximately the same values and varies from 1.4 to 2.4 nm (Figure S2)

**Figure S2.** (a) AFM topographic images of p-doped Y6 films with TrTPFB and with a thickness of 85nm; (b) root mean square roughness of the p-doped Y6 films measured on AFM.

**Section 3 – Organic solar cells measurements**

**Table S2.** Organic solar cell results for p-doping of Y6 with TrTPFB

| TrTPFB mol% | PCE, % (**max**) | J_sc_, mA/cm^2^ | V_oc_, V | FF, % |
| --- | --- | --- | --- | --- |
| 0 | 0.028 ± 0.008 (**0.04**) | 0.204 ± 0.034 | 0.39 ± 0.11 | 33.904 ± 3.44 |
| 0.56 | 0.062 ± 0.018 (**0.09**) | 0.466 ± 0.088 | 0.424 ± 0.103 | 31.339 ± 2.219 |
| 0.9 | 0.086 ± 0.026 (**0.12**) | 0.462 ± 0.078 | 0.562 ± 0.07 | 32.415 ± 2.502 |
| 5.06 | 0.372 ± 0.070 (**0.45**) | 1.792 ± 0.172 | 0.639 ± 0.102 | 32.554 ± 2.168 |
| 7.31 | 0.518 ± 0.142 (**0.67**) | 2.632 ± 0.384 | 0.536 ± 0.095 | 35.938 ± 1.007 |
| 9 | 0.398 ± 0.116 (**0.67**) | 2.276 ± 0.362 | 0.503 ± 0.088 | 34.41 ± 2.015 |
| 12.27 | 0.256 ± 0.076 (**0.37**) | 1.692 ± 0.372 | 0.49 ± 0.072 | 30.829 ± 2.617 |
| 15.84 | 0.358 ± 0.076 (**0.57**) | 2.476 ± 0.358 | 0.411 ± 0.04 | 35.038 ± 2.338 |
| 28.11 | 0.22 ± 0.06 (**0.37**) | 1.52 ± 0.29 | 0.494 ± 0.086 | 30.203 ± 2.498 |

**Section 4 – Charge transport measurements**

**Table S3.** Charge carrier mobility for holes and electrones for p-doping of Y6 with TrTPFB

| TrTPFB mol% | µ_h_, x 10^-4^ cm^2^/Vs | µ_e_, x 10^-4^ cm^2^/Vs |
| --- | --- | --- |
| 0 | 0.328 ± 0.064 | 0.22 ± 0.06 |
| 0.6 | 0.29 ± 0.074 | 0.235 ± 0.036 |
| 5.1 | 0.336 ± 0.069 | 0.237 ± 0.058 |
| 7.3 | 0.384 ± 0.083 | 0.273 ± 0.058 |
| 9.0 | 0.552 ± 0.154 | 0.152 ± 0.037 |
| 12.27 | 0.424 ± 0.077 | 0.291 ± 0.031 |
| 15.84 | 0.413 ± 0.053 | 0.36 ± 0.11 |
| 28.11 | 0.439 ± 0.108 | 0.418 ± 0.128 |

**Section 4 – Light-intensity dependent measurements**

**Table S5.** Ideality and “alpha” factors obtained from light-intensity dependent measurements of p-doped Y6 with TrTPFB OSC

| TrTPFB mol% | Ideality factor (light dependent) | Alpha factor (J_sc_ vs light intensity) |
| --- | --- | --- |
| 0 | 5.44 | 0.97 |
| 0.56 | 4.23 | 0.94 |
| 0.9 | 3.27 | 1.06 |
| 5.09 | 2.56 | 1.04 |
| 7.31 | 3.39 | 1.03 |
| 9.00 | 3.59 | 0.99 |
| 12.27 | 2.53 | 1.11 |
| 15.84 | 3.47 | 1.08 |
| 28.11 | 2.28 | 1.07 |

**Figure S3.** Light intensity-dependent *J-V* curves of OSC based on Y6 p-doped with a) 0 mol%; b) 0.56 mol%; c) 0.9 mol%; d) 5.1 mol%; e) 7.3 mol%; f) 9 mol%; g) 12.3 mol%; h) 15.8 mol%; i) 28.11% mol of TrTPFB.

**Section 5 – Density functional theory calculations**

**Figure S4.** DFT calculated HOMO/LUMO energy levels of Y6, Y6+, and [Y6-CP_3_]^*^.

**Section 6 – Transient absorption measurements**

As mentioned in section 1, TA measurements were performed using 515 nm excitation.

**Figure S5.** Spectral evolution of the transient absorption signal for (a) as-cast and (b) 5 mol% doped Y6 using a fluence of 6.0 µJ cm^-2^. (c) Comparative kinetics of the peak Y6 ground state bleach averaged from 850-870 nm using a fluence of 1.2 µJ cm^-2^.

**Figure S6.** dlog(J)/dlog(V) vs TrTPFB content in mol% in ohmic region of SCLC devices. Most of the fabricated SCLC devices are ohmic or near-ohmic.

**Figure S7.** Photoluminescence emission of Y6 thin films doped with TrTPFB.

**References**

1.	White, M. S., Olson, D. C., Shaheen, S. E., Kopidakis, N. & Ginley, D. S. Inverted bulk-heterojunction organic photovoltaic device using a solution-derived ZnO underlayer. *Appl. Phys. Lett.* **89**, 143517 (2006).

2.	de Mello, J. C., Wittmann, H. F. & Friend, R. H. An improved experimental determination of external photoluminescence quantum efficiency. *Advanced Materials* **9**, 230–232 (1997).

3.	Yanai, T., Tew, D. P. & Handy, N. C. A new hybrid exchange–correlation functional using the Coulomb-attenuating method (CAM-B3LYP). *Chemical Physics Letters* **393**, 51–57 (2004).

4.	Kronik, L., Stein, T., Refaely-Abramson, S. & Baer, R. Excitation Gaps of Finite-Sized Systems from Optimally Tuned Range-Separated Hybrid Functionals. *J. Chem. Theory Comput.* **8**, 1515–1531 (2012).

5.	Sun, H. *et al.* Ionization Energies, Electron Affinities, and Polarization Energies of Organic Molecular Crystals: Quantitative Estimations from a Polarizable Continuum Model (PCM)-Tuned Range-Separated Density Functional Approach. *J. Chem. Theory Comput.* **12**, 2906–2916 (2016).

6.	Refaely-Abramson, S. *et al.* Gap renormalization of molecular crystals from density-functional theory. *Phys. Rev. B* **88**, 081204 (2013).

7.	Chen, X. & Brédas, J. Voltage Losses in Organic Solar Cells: Understanding the Contributions of Intramolecular Vibrations to Nonradiative Recombinations. *Advanced Energy Materials* **8**, 1702227 (2018).

8.	M. J. Frisch, G. W. Trucks, H. B. Schlegel, G. E. Scuseria, M. A. Robb, J. R. Cheeseman, G. Scalmani, V. Barone, G. A. Petersson, H. Nakatsuji, X. Li, M. Caricato, A. V. Marenich, J. Bloino, B. G. Janesko, R. Gomperts, B. Mennucci, H. P. Hratchian, J. V. Ortiz, A. F. Izmaylov, J. L. Sonnenberg, D. Williams-Young, F. Ding, F. Lipparini, F. Egidi, J. Goings, B. Peng, A. Petrone, T. Henderson, D. Ranasinghe, V. G. Zakrzewski, J. Gao, N. Rega, G. Zheng, W. Liang, M. Hada, M. Ehara, K. Toyota, R. Fukuda, J. Hasegawa, M. Ishida, T. Nakajima, Y. Honda, O. Kitao, H. Nakai, T. Vreven, K. Throssell, J. A. Montgomery, Jr., J. E. Peralta, F. Ogliaro, M. J. Bearpark, J. J. Heyd, E. N. Brothers, K. N. Kudin, V. N. Staroverov, T. A. Keith, R. Kobayashi, J. Normand, K. Raghavachari, A. P. Rendell, J. C. Burant, S. S. Iyengar, J. Tomasi, M. Cossi, J. M. Millam, M. Klene, C. Adamo, R. Cammi, J. W. Ochterski, R. L. Martin, K. Morokuma, O. Farkas, J. B. Foresman, and D. J. Fox, Gaussian~16 Revision C.01 (2016), Gaussian Inc. Wallingford CT.
